# Botanical Description of British Plants

**Published:** 1806-08

**Authors:** 


( To be continued. )

---

### *Botanical Description of British Plants.*

[ Continued from our last, p. 66—72. ]

2. PRUNUS. *P. cerasus.*
*Ang.* Common wild cherry-tree.
*Gen. Desc.* *As above.*

*Spec. Desc.* *Umbels* mostly on short fruit-stalks. *Leaves* egg-spear-shaped, smooth, doubled together. *Leaf-scales* toothed. *Floral-leaves* three-cleft, serrated. *Leaf-buds* terminating; *fl. buds* lateral. *Bloss.* white. *Fruit*, red. *Woods, hedges.* Bl. May.

*Use.* The common people eat the fruit either fresh or dried; and it is frequently infused in brandy for the sake of its flavor. The gum that exudes from the trunk and branches, is equal to gum arabic. Hasselquist relates that, during a seige, more than an hundred men were kept alive for two months nearly, without any other sustenance than a little of this gum taken into the mouth sometimes, and suffered gradually to dissolve.—The wood is hard and tough; it is used by the turner, and is much employed for making chairs, and for staining in imitation of mahogany. This tree, which is the original stock whence many of the cultivated kinds are derived, loves a sandy soil, and an elevated situation.—*Linn.*

3. Prunus. *P. domestica*,—*P. gallicum. P. vulgaris.*
*Ang.* Common plumb-tree.
*Gen. Desc.* *As above.*

*Spec. Desc.* *Fruit-stalks* mostly solitary. *Leaves* egg-spear-shaped, coiled. *Branches* thornless. *Flower-buds* producing no leaves. *Cal.* sometimes six-cleft. *Style* crooked. *Hedges.* Bl. April.

*Use.* From this species the varieties of the garden plumb are derived by culture.—The bark dyes yellow.—It loves a lofty exposure, and is favourable to pasturage.—*Withering.* When perfectly ripe and eaten in a moderate quantity, all the garden plumbs are a pleasant and wholesome food. But in an immature state they are more liable to produce colicky pains, diarrhœa, or cholera, than any other fruit of this class; attention to this circumstance is therefore necessary. Considered medicinally, they are *emollient, cooling,* and *laxative,* especially the French prunes, which are imported dried from Marseilles: and though the purgative power of these is diminished by drying, yet, it is observed by Dr. Cullen, that as they contain much of the acid which they originally had, they have more effect in this way, than the other dried fruits. *M. M.* 2. 254. They are found peculiarly useful in costive habits, and are frequently ordered in decoction with senna, or other purgatives. It is the pulp of this fruit which is directed in the lenitive electuary, or elect. è senna.—*Woodville.*

4. Prunus.

4. Prunus. *P. Insititia.*

*Ang.* Bullace plumb. Black bullace tree.

*Gen. Desc.* *As above.*

*Spec. Desc.* *Fruit stalks* in pairs. *Leaves* egg-shaped, slightly woolly, coiled. *Branches* reddish brown, smooth, ending in a thorn. *Stipulæ* narrow, fringed, cloven, sharp. *Cup* smooth. *Style* straight. *Flowers* white. *Fruit* black. *Hedges.* Bl. April.

*Use.* An infusion of the flowers sweetened with sugar is a mild *purgative*, not improper for children. The bark of the roots and branches is considerably *styptic.*—The fruit is acid, but so tempered by a sweetness and roughness, as not to be unpleasant, particularly after it is mellowed by the frosts. A conserve is prepared by mixing the pulp with thrice its weight of sugar.—*Withering.*

5. Prunus. *P. spinosa.*—*P. sylvestris.*

*Ang.* Black thorn. Sloe tree. Scroggs.

*Gen. Desc.* *As above.*

*Spec. Desc.* *Branches* thorny. *Fruit stalks* solitary. *Leaves* spear-egg-shaped, smooth, serrated; the serratures terminated by an excretory duct. *Leaf-scales* strap-shaped, toothed, points of the teeth as if dead. *Styles* sometimes 2. *Hedges.* Bl. March, April.

*Use.* The pruna sylvestria have been employed for their *styptic* power since the time of Dioscorides; and as their astringency is united to the refrigerant qualities of the fruit, they may sometimes supersede those medicines of this class which are of a resinous or heating quality. They have been recommended in *diarrhœas, hæmorrhagic* affections, and as gargles in *tumefactions of the tonsils* and *uvulæ.* Dr. Cullen considers the sloe as the most powerful of the *fructus acerbi,* and adds that he has often found it an agreeable and useful *astringent.* The flowers with their calyces are moderately *purgative,* and for this purpose one oz. infused in a sufficient quantity of whey, was found to be a pleasant and useful laxative. The powdered bark, in doses of one drachm, is said to cure *agues.*—*Woodville.* An infusion of a handful of the flowers is a safe and easy purge. The bark powdered, in doses of 2 drachms, will cure some agues.—*Withering.* The fruit or sloe is so sharply harsh and austere as not to be eatable till thoroughly mellowed by frosts. The juice is extremely viscid, so that the fruit requires the addition of a little water in order to admit of expression: the juice obtained from the unripe fruit, and inspissated to dryness by a gentle heat, is the German acacia of the shops, and has often been sold for the Egyptian acacia.—*Woodville.* The fruit bruised and put into wine gives it a beautiful red colour, and a pleasant subacid roughness. The tender leaves dried, are sometimes used as a substitute for tea; and it appears to be the best substitute that has yet been tried. From effects which Dr. W. has repeatedly observed to follow from the prick of the thorns, he concludes that they contain something poisonous, particularly in autumn. Letters written with the juice of the fruit, upon linen or woollen, will not wash out. The wood is hard, tough, and is used for the teeth of rakes, and for walking sticks. This tree is not well adapted to grow in hedges, because it spreads its roots wide, and encroaches upon the pasturage; but it makes a good dead fence. Horses, sheep and goats, eat the leaves. —*Withering.*

Icosandria. Digynia.

6. CRATÆGUS. *C. aria.*

*Anj.* White-beam hawthorn. White-beam tree. Wild pear tree.

*Gen. Desc.* Cal. 5-cleft. Pet. 5. Berry beneath, opening at top, 1 celled, 1 or more seeded.

*Spec. Desc. Leaves* egg shaped, cut, serrated, cottony underneath. *Cal.* woolly. *Petals* scolloped, woolly at the base, white. *Styles* 2 or 4, woolly at the base. *Seeds* 2 in each cell. *Fruit* red. *Woods, hedges, especially in mountains and in calcareous soil.* BL. May.

*Use.* The fruit is palatable when mellowed by the autumnal frost; and from it an ardent spirit may be distilled. The wood is hard, tough and smooth; used for axle-trees, wheels, walking-sticks, carpenter's tools, &c. It affords an excellent charcoal for the manufacture of gunpowder. It loves dry hills and open exposures; flourishes either in gravel or clay; bears lopping, and permits the grass to grow under its shade; but it rarely bears a good crop of fruit two years successively. Sheep and goats eat it greedily.—*Withering.*

Icosandria. Trigynia.

7. SORBUS. *S. aucuparia.*

*Ang.* Quicken-tree. Mountain ash. Roan tree. Service.

*Gen. Desc.* Cal. 5 cleft. Petals 5. Pomum 5 celled, open at the top, 3 seeded.

*Spec. Desc. Leaves* winged, smooth on both sides, rib channelled. *Leafits* 7 or 8 pair sitting, spear-shaped, serrated.

rated. *Corymbus* terminating. *Berry* round, scarlet. *Seeds* 3, 4, 5, reddish. *Flowers* whitish. *Woods, hedges, mountains, boggy situations.* Bl. April.

*Use.* The berries infused in water produce an acid liquor, somehat like perry, which is used for drink by the poorer people in Wales. Dried and reduced to powder, the berries make a wholesome bread; and an ardent spirit may be distilled from them, which has a fine flavour, but is small in quantity. In Germany the berries are used by fowlers to entice the red wings and fieldfare into nooses of hair suspended in the woods; hence its trivial name. The wood is soft, tough, and solid; it is used for making tables, chairs, wheelspokes, shafts, &c.; and the roots are formed into knife handles, and wooden spoons. It grows either in woods or open fields, but best on the sides of hills, and in fertile soil; it will not bear lopping; and plants grow well in its shade.—*Withering.*

8. Sorbus. *S. domestica.*

*Ang.* True service, or sorb.

*Gen. Decs.* *As above.*

*Spec. Desc.* *Leaves* winged, woolly underneath. *Bloss.* white. *Fruit* brownish. the size of a crab; cells 5; seldom perfects all the seeds. *Mountainous forests.* Bl. Apr.

*Use.* The fruit is mealy and austere, not much unlike the medlar. The wood is particularly valuable for making mathematical rulers and excisemen's gauging-sticks.—*Withering.*

Icosandria .Pentagynia.

9. Mesphilus. *M. germanica.*

*Ang.* Medlar tree.

*Gen. Desc.* Cal. 5 cleft. Petals 5. Berry with 1, 2, or 5 cells.

*Spec. Desc.* Thornless, woolly. *Leaves* oval-spear shaped, cottony underneath; towards the point serrated. *Leaf-stalks* short, channelled. *Flowers* solitary, sitting. *Cal.* terminating, hairy, fleshy, woolly within. *Stamens* unequal, 30 or more. *Summit* cloven. *Bloss.* white. *Fruit,* reddish brown. *Hedges about Minchiville.* Bl. May.

*Use.* The fruit, when it becomes soft by keeping, is very palatable, and many peeple are fond of it; but it is somewhat austere, and binding to the bowels.—*Withering*

10. Pyrus. *P. Communis.*

*Ang.* Pear-tree.

*Gen. Desc.* *Cal.* 5 cleft. Petals 5. Pornum beneath 5 celled, many-seeded.

*Spec.*

*Spec. Desc. Leaves* serrated, smooth. *Flowers* in a corymbus, white. *Woods.* Bl. April, May.

*Use.* The fruit is austere, but when improved by cultivation, highly grateful, as is proved by the great variety of excellent pears, which the industry of mankind has raised, for from this they all originate. The juice of the fruit fermented is commonly used as a beverage, known by the name of perry, which is chiefly made in Worcestershire and Herefordshire. The Squash, Oldfield, and Barland perrys are reckoned the best, and are little inferior to wine. The leaves afford a *yellow* dye, and may be used to give a green to blued cloths. The wood is light, smooth and compact; it is used by turners, and for making joiner's tools: of it also are made picture frames, which are to be stained black. It loves a fertile soil and sloping ground, but will not thrive in moist bottoms. It stands the severest winters, and does not destroy the grass. Horses, cows, sheep and goats eat the leaves.—*Withering.*

Pyrus. *P. malus.*

*Ang.* Crabtree, or wilding. *Cultivated var.* Apple-tree.

*Gen. Desc. As above.*

*Spec. Desc. Leaves* serrated, more circular than the P. communis. *Flowers* in umbels, sitting; *petals* white, tinged with red outside. *Woods, hedges, or cul. in orchards, hedge-rows.* Bl. May.

*Use.* The acid of the juice of the crab or wilding is called by the country people verjuice, and is much used in recent *sprains,* and in other cases, as an *astringent* or *repellent.* With a proper addition of sugar, it is probable that a very grateful liquor might be made of this juice, but little inferior to old hock. The bark affords a yellow dye. The wood is tolerably hard; it turns very clean, and cogs of wheels made of it obtain a polish, and wear a long time. It flourishes better on declivities and in shady places, than in open exposures or boggy situations; grass and even corn will grow under its shade. It is much used as a stock on which to graft the better kinds of apples, because its roots are neither killed by the frost, nor eaten by field mice. Horses, cows, sheep and goats eat the leaves; swine are very fond of the fruit.

The juice of the cultivated apple, fermented, is well known by the name of cyder, of which large quantities are made in Herefordshire, Devonshire, part of Worcestershire and Gloucestershire, and in the south of Ireland, in a soil of deep clay. The stronger sorts, as the Styre cyder, cyder, will bear exportation to the East or West Indies. The cyder apple-trees were originally brought from Normandy; and it is supposed by many, that the liquors would be now improved by a fresh importation.—*Withering.*

12. Spiræ. *S. ulmaria.*

*Ang.* Meadow sweet. Queen of the meadows.

*Gen. Desc.* *Cal.* five-cleft. Petals 5. Caps. 4 or more, two-celled, 2 valved, many-seeded.

*Spec. Desc.* *Stem* angular, reddish. *Leaves* interruptedly winged; *leafits,* egg-shsped, doubly, but irregularly serrated, hoary underneath, bright green, terminating leaf three-segm. *Flowers* yellow white. *Cal. segm.* and *pet.* sometimes 4; caps. mostly 6, twisted. *Moist meadows.* Bl. June, July.

*Use.* The whole plant has an *astringent* quality, and as such has been found useful in *dysenteries* and *ruptures.* A distilled water from the flowers has great efficacy in expelling the *measles* and *small-pox.* This plant has been used for tanning leather.—*Lightfoot.* The flowers infused in boiling water, give it a fine flavour, which rises in distillation. Goats are extremely fond of it; sheep and swine eat it; horses and cows refuse it.—*Withering.*

13. Spiræa. *S. filipendula.*

*Ang.* Dropwort. Meadow sweet.

*Gen. Desc.* *As above.*

*Spec. Desc.* *Stem* herbaceous. *Leaves* interruptedly winged; *leafits* strap spear-shaped, irregularly serrated, very smooth on both sides, mostly alternate; a pair of little leafits on the leaf stalk between each larger pair. *Flowers* in tufts. *Fruit stalk* crooked before the flowers expand. *Petals* cream colour, purplish underneath, turned back. *Styles* many. *Caps* numerous, disposed in a circle. *Mountainous, meadows in calcareous soil.* Bl. June, July.

*Use.* The tuberous pea-like roots, dried and reduced to powder, make a bread, which in times of scarcity is by no means to be despised. Hogs are very fond of the roots.—*Linn.* When expanded and enlarged by cultivation, this plant is a beautiful addition to the flower garden.—*Withering.*

